# The Bran and Grain Grinding Level Affect the Tensile Characteristics of Bioplastics Derived from Wholegrain Wheat Flours

**DOI:** 10.3390/polym15224381

**Published:** 2023-11-10

**Authors:** Franco Dominici, Debora Puglia, Francesca Luzi, Catia Governatori, Giacomo Tosti, Paolo Benincasa

**Affiliations:** 1 Civil and Environmental Engineering Department, University of Perugia, UdR INSTM, 05100 Terni, Italy; franco.dominici@unipg.it; 2 Department of Science and Engineering of Matter, Environment and Urban Planning (SIMAU), 60131 Ancona, Italy; f.luzi@staff.univpm.it; 3 AMAP, Agenzia Marche Agricoltura Pesca, Via Giulio Latini, 64, 60035 Jesi, Italy; governatori_catia@amap.marche.it; 4 Department of Agricultural, Food and Environmental Sciences, University of Perugia, Borgo XX Giugno, 74, 06124 Perugia, Italy; giacomo.tosti@unipg.it (G.T.); paolo.benincasa@unipg.it (P.B.)

**Keywords:** *Triticum aestivum*, milling, Chopin’s alveograph, plasticization, stress–strain curve, compost, phytotoxicity, germination

## Abstract

The mechanical performance of thermoplastic bulk samples obtained by plasticizing wheat flours differing in grain hardness, alveographic parameters, absence or presence of bran, and grinding level was assessed. Grains of four bread wheat (*Triticum aestivum* L.) cultivars (Altamira, Aubusson, Blasco, and Bologna) were milled with the aim of producing single-cultivar refined flour (R), or wholegrain flour with fine (F) or coarse (C) grinding. The flours were plasticized, injection molded and tested for tensile properties. The results confirmed that the presence of bran increased the strength (σ) and reduced the elongation at break (ε) of thermoplastics obtained from the flours of each cultivar. The grinding level had an effect, since σ was higher and ε was lower in F than in C samples. SEM analysis of samples revealed that the bran and its texture affected the exposure of starch granules to plasticizer. Composting experiments also revealed that the formulations are able to disintegrate within 21 days with a mass loss rate higher in plastics from F than C flours, while germination tests carried out with cress seeds indicated that it takes two months before the compost loses its phytotoxic effects. Overall, the refining and bran particle size of wheat flours, besides their gluten composition and baking properties, represent novel choice factors to be considered when tailoring the manufacturing of plastic materials for selected requirements and uses.

## 1. Introduction

The increasing cost of petrol-based plastics and the public concern about their contribution to environmental pollution have been increasing interest towards biobased materials, since these are biodegradable and help dispose of by-products from the agricultural production and food industries. In the case of bioplastics, purified starch from many agricultural sources (e.g., cereals, tubers, etc.) is often used as basic ingredient. However, there is literature on the use of wheat flours to obtain bioplastics as an energetically and economically cheap alternative to purified starch [1].

Previous research from our group demonstrated that the tensile properties of thermoplastic films may depend on wheat grain hardness and baking properties of refined flours as affected by the wheat genotype [2,3], or by cultivation practices, such as nitrogen fertilization [4]. These properties may be quantified by making a dough with standard procedure from wheat flour and water, and testing dough deformation using Chopin’s alveograph, where dough disks are blown by an air flow until bubble explosion [5]. In the outcoming alveogram, the maximum value in the *y*-axis represents the maximum pressure (P) needed to start blowing the dough disk, which expresses the tenacity of the dough; the maximum value on the *x*-axis is the time needed to blow up the bubble, which expresses the extensibility of the dough (L); the P/L ratio indicates the curve shape; the area below the curve, i.e., its width (W), expresses the dough deformation energy.

To date, few studies have considered the use of wholegrain flours, while this could be of relevance because the bran could work as reinforcement for the starch-based matrix [6,7]. The bran represents the outer portion of the grain, including the pericarp and seed teguments, containing a relevant amount of cellulose and some lignin, the so-called fiber. It accounts for around 15–25% of the total grain weight and generally comes out as a by-product of grain milling, representing the grinding tail. It is normally used as animal feed, However, the progressive decrease in the whole national livestock occurred in the last dozens of years have implied an increase in bran stocks to eliminate, with thermovalorization as a main alternative [8,9]. 

The use of bran as reinforcement in thermoplastics has been little explored: in their paper, Aliotta et al. [10] investigated the use of wheat bran (WB) as a potential filler for influencing the plasticizer migration in poly(lactic acid) (PLA)/poly(butylene succinate adipate) (PBSA) binary blends (with 60 wt.% of PLA and 40 wt.% of PBSA). Strangis et al. [11] focused on the development and characterization of biocomposites based on poly(butylene succinate-co-butylene adipate) and wheat bran (5, 10, 15, and 20 wt.%) derived by flour milling. The authors observed that, in marine biodegradation tests, the bran had the capacity to improve the PBSA biodegradation rate, probably due to the hygroscopic bran swelling. Dominici et al., also found that the bran % content in wholegrain flours of bread wheat may affect the tensile properties of thermoplastics [12]. Combrzyński et al. observed that the amount of durum wheat bran additive used, as well as the temperature of high-pressure injection molding, both had a significant impact on the structure and mechanical properties of the TPS moldings subjected to mechanical strength testing [13]. Robin and coauthors also found that bran concentration influenced the glass transition temperature, melting temperature and sorption isotherm of the unprocessed wheat flour, that might contribute to the modulation of the expansion properties of a bran-containing starchy foam [14].

In addition, the bran particle size, as affected by the grain grinding level, is likely to affect the mechanical properties of thermoplastics but, to date, no research has investigated this aspect. Bran can act as an effective natural reinforcement for plasticized flours by realizing a sort of thermoplastic biocomposite. As previously observed for the effect of bran amount in plasticizable mixtures, the size of the fibers can also have different impacts [12]. Fine-sized bran particles can be better dispersed within the biocomposite, reinforcing and blocking the plastic phase, while coarse bran fibers can be the site of concentrated stress, providing substantial reinforcement on the one hand, but also causing defects at the interface with the matrix, which act as a trigger point for fractures. 

Typically, plasticized flours reach 90% wt. of disintegration into compost after 2–3 weeks. In other studies, Dominici et al. observed that the presence of bran slowed down the degradation kinetics during the first week [12]. In this time interval, the more complex and stable components present in bran fibers were reduced to simpler carbohydrates and sugars [15], while, in the following weeks, the disintegration kinetics became comparable to the one observed for plasticized refined flours. It can be assumed that the markedly biodegradable materials studied in this work can reach disintegration into compost in 2–3 weeks, significantly less than the 90 days required by law [16]. Hence, by considering that even if the literature already considered the use in bran in commercial bioplastics and its grinding level, the literature lacks in the definition of the pivot role of flour refining for plasticization purposes. On the basis of these considerations, the purpose of this study was to describe the mechanical performance of thermoplastic bulk samples obtained from bread wheat flours differing for source grain hardness, alveographic parameters, and refining and bran grinding level. The plastic composting process of the different samples and the compost phytotoxicity were also evaluated. 

## 2. Materials and Methods

Flours: Grains of four bread wheat (*Triticum aestivum* L.) cultivars (Altamira, Aubusson, Blasco, and Bologna) were milled separately by AMAP (Jesi, Ancona Province) by using a laboratory mill (Labormill, 4.RB, R. Bona SRL, Monza, Italy) with a standard procedure to obtain refined flours (R), and another laboratory mill (Bühler MLI-204, Bühler AG, Uzwil, Switzerland) to obtain wholegrain flours, which was set at two extremely different grinding levels, 0 and 9, corresponding to very fine (F) and very coarse (C) grinding. The four cultivars were selected among the 26 considered by Benincasa et al., taking into consideration the different grain hardness of the cultivars and the different alveographic characteristics (P, L, P/L, W) of the refined flours and tensile properties of the derived thermoplastic films (strength, σ; elongation at break, ε) [3]. These characteristics depend primarily on the genotype but can vary year by year according to environmental conditions (i.e., mainly soil properties and season climate) and cultivation practices. For this reason, to guarantee the same flour characteristics, the same flour samples used by Benincasa et al. were used here, i.e., these flours were obtained from the same source grains collected in the same field experiment and year, and milled with the same laboratory mill [3]. Therefore, the characteristics of the four refined flours reported in Table 1 are the same as in Benincasa et al., [3] and were obtained as detailed therein, by using a Chopin’s alveograph (Alveolink NG, Tripette & Renaud, Villeneuve la Garenne, France) in constant hydration (HC) mode, in agreement with the recommendations of the ISO 27971 standard [17]. The alveographic parameters P (maximum overpressure), L (abscissa at rupture), W (deformation energy), as P/L (configuration ratio) were obtained as the average values of the five alveograms produced from five doughs.

**Figure 1:**
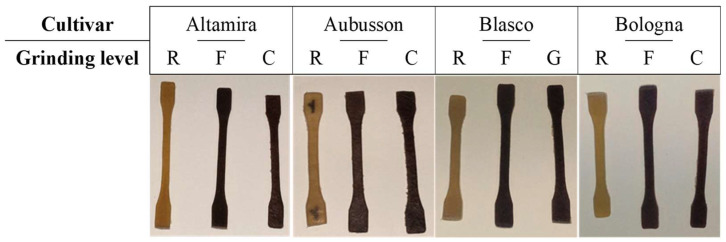
Samples of biocomposites for tensile tests based on plasticized flours of the four cultivars. The samples of each cultivar were obtained by plasticizing a refined flour (R) and two wholegrain flours with fine (F) or coarse (C) grinding.

Production of thermoplastic bulk samples: The flours were plasticized to produce bulk samples for the characterization (Figure 1). After drying the flours in a vacuum oven at 40 °C for 48 h, the dough for whole grain flours F and C was prepared with 75% wt. of flour, adding 10% wt. glycerol and 15% deionized water as plasticizers and kneading in a planetary mixer for 15 min. This formulation was simplified compared to our previous works, with the aim of producing a simple and cost-effective biomaterial for injection molding [2,3,12]. The formulation of refined flour R was appropriately modified in consideration of the different bran fiber and plasticizable content compared to wholegrain flours. To plasticize the dough, an extruder/compounder (DSM Xplore 5 & 15 Micro Compounder, Xplore Instruments BV, Sittard, The Netherlands) was used, with a double co-rotating screw (Screw length 337 mm, mixing section length 172 mm with conical shape of diameter from 22.1 to 8.9 mm) at a speed of 30 rpm and with a temperature profile of 130, 135 and 140 °C for the feeding, metering and die zones; this compounder was equipped with a flow diverter which allows the recirculation of melted polymer for a certain time or exit through the die. An injection moulder (DSM Xplore Micro 10cc Injection Moulding Machine, Xplore Instruments BV, Sittard, The Netherlands), coupled to the extruder, was employed to produce the specimens of the twelve materials to be tested (Figure 1). The compounded materials were then forced into a closed mould with a dumbbell-shape (1BA type geometry, compliant with the ISO 527-2 standard).

Thermogravimetry: The thermal degradation of the twelve wheat flours, obtained from four bread wheat varieties subjected to three different milling procedures (R, F, C), was evaluated performing a dynamic thermal test, from 30 °C to 900 °C by thermogravimetric analyzer (TGA, Seiko Exstar 6300, Tokyo, Japan). About 10 mg of each sample was used, and dynamic tests were performed under nitrogen flow (200 mL·min^−1^). At the constant heating rate of 10 °C min^−1^, the mass loss (TG) and mass loss rate (DTG) curves of each flour were evaluated.

Differential Scanning Calorimetry: The thermal behavior of wheat flours, after mixing with the plasticizers, was studied using a Differential Scanning Calorimeter Q200 (DSC TA instruments, New Castle, DE, USA). After the calibration of the instrument with indium standard, about 10 mg of each dough sample was placed in a sealed sample crucible. DSC scans were performed in the temperature range from 25 to 180 °C, obtaining thermograms that report the heat flow necessary to increase the temperature of each sample of unit mass at a heating rate of 10 °C min^−1^. 

Morphological analysis: The morphological characterization of each of the twelve flours (four varieties by three millings), and their plasticized bulk samples was carried out using a Supra 25 field emission scanning electron microscope (FESEM) from Zeiss (Oberkochen, Germany). The flours samples were deposited onto a conductive adhesive tape substrate and coated with gold sputter to provide electrical conductivity. The plasticized samples were cryofractured in liquid nitrogen, and the resulting surfaces coated by gold sputtering. SEM micrographs of the fracture surfaces were taken with an accelerating voltage of 5 kV at different magnifications.

Moisture content: The plasticized samples were maintained for 48 h in controlled environmental conditions at a temperature of 20 °C with relative humidity (RH) of 53%. Three samples of each formulation were initially weighed, then placed in a vacuum oven at 60 °C and weighed every 24 h until weight consistency was achieved. The weight change caused by moisture loss and the average percentage weight change in each material were calculated (Equation (1)):

(1) $$M_{c}=\frac{\left(M_{0}-M_{d}\right)\times 100}{M_{0}}$$

M_c_ = Moisture content% wt. M_0_ = Mass of initial wet sample. M_d_ = Mass of the dried sample.

Water uptake and mass loss: The absorption tests were carried out on samples conditioned in controlled environments for 48 h at a temperature of 20 °C with a relative humidity of 53%. Initially, three samples for each type of flour were weighed. They were subsequently placed in containers with deionized water and left completely immersed for a day. The samples, taken from the water, were lightly buffered to remove excess surface humidity and were weighed again after absorption to obtain the absorption by weight of water Ma (Equation (2)).

(2) $$M_{a}=\frac{\left(M_{as}-M_{bs}\right)\times 100}{M_{bs}}$$

M_a_ = Mass of absorbed water% wt. M_as_ = Mass of the sample after soaking. M_bs_ = Mass of the sample before soaking.

Subsequently, the samples were dried in an oven at 60 °C for 24 h and subjected to conditioning for 48 h with the same starting environmental conditions. The samples were weighed again, and the mass loss M_r_ caused by the release of substances during immersion in water was calculated (Equation (3)).

(3) $$M_{r}=\frac{\left(M_{asdc}-M_{bs}\right)\times 100}{M_{bs}}$$

M_r_ = Mass released after soaking% wt. M_as_ = Mass of the sample after soaking, drying and conditioning. M_bs_ = Mass of the sample before soaking.

The real mass loss (%wt.) was calculated by subtracting the moisture content M_c_, reabsorbed after the last environmental conditioning, from the mass lost due to release M_r_, according to the equation M_rr_ = M_r_ − |M_c_|. Similarly, the water really absorbed (%wt.) was calculated by adding to the absorbed water M_a_ also the mass lost due to release M_r_, replaced by water, according to the equation M_ar_ = M_a_ + |M_r_|.

Mechanical Characterization: The mechanical characterization of the thermoplastic samples obtained from each of the twelve flours was carried out with tensile tests performed by a universal electronic dynamometer LR30K Plus (Lloyd Instruments, Bognor Regis, UK), which can operate with different load cells from 5 N up to at 30 kN, according to the ISO 527 standard [18]. After conditioning in a controlled environment at 20 °C with 53% relative humidity for 48 h, at least 5 samples for each formulation were subjected to traction at a crosshead speed of 5 mm·min^−1^ and the values of the resistant force were obtained as a function of the gap between the clamps. The values of Young’s modulus (E), tensile stress (σ) and strain at break (ε) were calculated and the stress–strain (σ–ε) characteristic curves were plotted with the support of specific software for the dynamometer (NEXYGEN Plus Materials Testing, Lloyd Instruments, Bognor Regis, UK).

Composting experiment: The compost mineralization of Altamira series (taken as a reference) was evaluated on the basis of the ISO 20200 standard [19]. A certain amount of compost inoculum, supplied by Gesenu Spa, was mixed together with synthetic organic waste, prepared with an appropriate amount of sawdust, rabbit feed, starch, sugar, oil and urea to thus constitute the soil for composting. The soil moisture content was maintained at values of 50% RH by adding water and mixing at regular intervals of time, as indicated by the legislation, while aerobic and thermal conditions were guaranteed during the test. Based on ISO 20200, a sample can be considered disintegrated when it reaches 90% mass disintegration in at least 90 days in contact with the composting soil in the ripening phase. The disintegration percentage (D_t_) after a time t in compost was calculated as the difference between the initial mass of the sample (M_i_) and the mass of the extracted sample, after drying, at a given time t (M_t_) (Equation (4)):

(4) $$D_{t}=\frac{\left(M_{i}-M_{t}\right)\times 100}{M_{i}}$$

Evaluation of compost phytotoxicity: The phytotoxicity of composts was evaluated by using the composts obtained from the plastic bulk samples R, F and C of cultivar Altamira. To this aim, the germination performance of cress (*Lepidum sativum* L.) seeds on a substrate soaked with a water extract of compost was determined with the same procedure adopted in Dominici et al. [12]. In detail, each compost sample (200 g) was watered until 85% *w*:*w* and left for 2 h, then centrifuged at 6000 rpm for 15 min and the surnatant was filtered under a 3.5 atm pressure by a sterilizing membrane. The water extracts were then diluted at 75 and 50% and used to soak the Whatman paper contained in Petri dishes of 9 cm diameter, where 10 cress seeds, previously soaked in distilled water for 1 h, were then positioned. Petri dishes were then incubated at 27 °C for 24 h, according to a randomized block design with four replicates (Petri dishes). After this time interval, germinated seeds were counted and seedling root length was measured. The germination index (GI) was calculated as (Equation (5)):

(5) $$GI\%=\frac{\left(GNc*Lc\right)\times 100}{GNt*Lt}$$

GNt = average number of germinated seeds in each treatment. GNc = average number of germinated seeds in the control. Lt =average root length of each treatment. Lc = average root length in the control.

## 3. Results and Discussion

Alveographic characteristics: The four bread wheat varieties tested here were chosen for their very different alveographic characteristics [20,21], as reported in Benincasa et al. [3], in order to obtain generalizable information on the effect of wholegrain grinding on the mechanical properties of plasticized flours. In particular, Altamira was a soft grain cultivar and Aubusson was a hard grain cultivar having soft-like characteristics (i.e., low values of W, P and P/L, and high values of L), while Blasco and Bologna were typical hard grain cultivars with high W, but differing in P, L and P/L values (Table 1).

**Figure 2:**
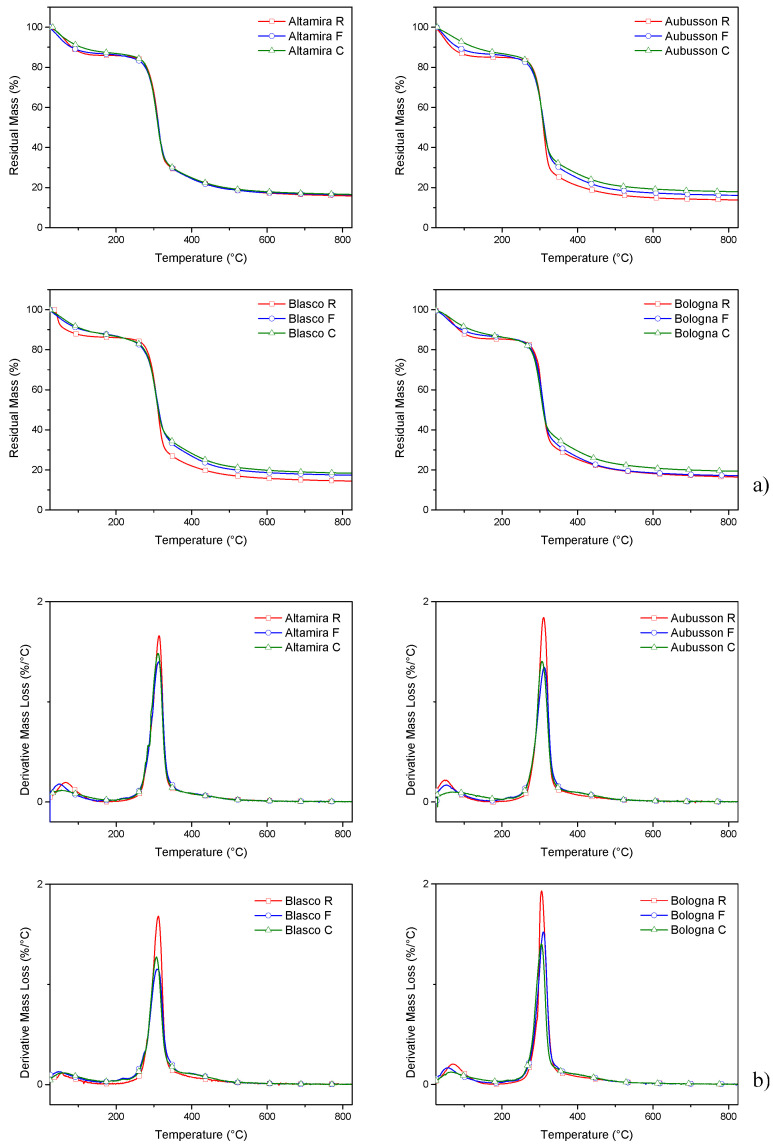
Thermogravimetric curves of flours from the four bread wheat varieties used for plasticization. The flours were refined (R) or wholegrain with fine (F) or coarse (C) grinding: (**a**) mass loss and (**b**) derivative mass loss.

Thermal analysis: The thermogravimetric analysis evidenced the typical degradative TGA pattern (Figure 2a) for cereal flours, with four main peaks in derivative DTG (Figure 2b). The first weight loss between 12 and 15% wt. is due to the evaporation of humidity around 100 °C. Water loss was generally slightly more intense for refined flours (14–15% wt.) while wholegrain flours showed slower release and at lower temperatures, due to the bran fiber content. The shape of this low temperature peak appears asymmetrical, due to the different water release dynamics in relation to the content and characteristics of the bran [12,22]. A second peak between 200 and 250 °C, more evident for wholegrain flours, represents the beginning of the decomposition of the lignocellulosic components (mainly hemicellulose and cellulose) contained in the bran fraction. This second peak is partially overlapped by the more intense degradation peak at 300–315 °C, represented mainly by the degradation of starch. This main peak shows a shoulder at higher temperatures, even above 400 °C, attributable to the degradation of the more thermally stable lignocellulosic components (mainly lignin) and the formation of inert carbonaceous residues [1,23]. There is a lesser differentiation in the residual mass between refined flour and wholegrain flours for the soft cultivar (Altamira) when compared to the grain hardness of hard cultivars: this result can be related to the composition of the pericarp, the texture of the endosperm and, therefore, to the different ratios of the bran components [24,25]. Indeed, grain hardness has been found to be associated also to a different composition of the outer grain layers, namely to the content of cellulose, arabinoxylans, β-glucans, etc., as affected primarily by the cultivar [26].

**Figure 3:**
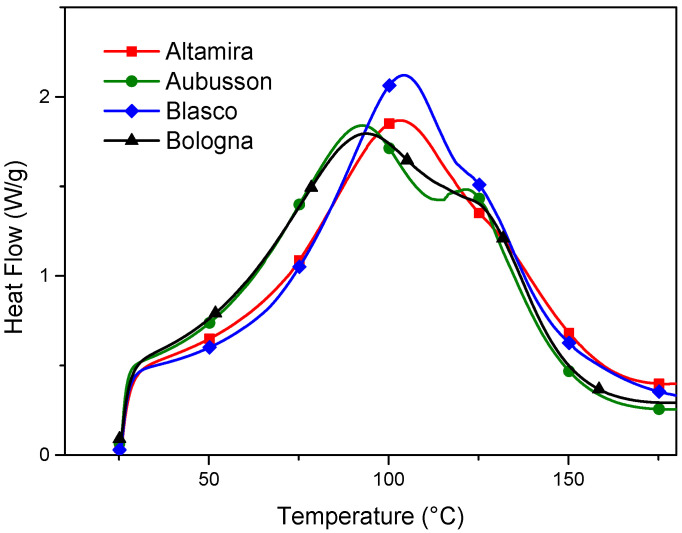
DSC thermogram of gelation/plasticization of doughs obtained from refined flours of four bread wheat cultivars with water and glycerol.

The DSC analysis was carried out to define the thermodynamics of gelation of the flours and therefore the range of temperatures suitable for the plasticization of the ternary doughs (flour/water/glycerol) to be set in the extruder. The DSC thermograms (Figure 3) show that, already at low temperatures, the start of the gelatinization reaction is detected, which is attributable to the rearrangement of amylose in the amorphous domains of the starch granules. At lower temperatures, during the first part of the starch gelatinization/melting process, the water absorbed by the granules increases the mobility, especially of amylose, in the amorphous domains, leading to realignment and the formation of new intermolecular bonds [27,28]. As the temperature increases, plasticization with glycerol is also activated, producing the superposition of the transformation peaks of the two-plasticizer system and adding their enthalpies. Depending on the composition of the starch, and in particular the characteristics of the amylose, different peak shapes are noted for the different flours. In particular, for Aubusson, the two peaks are distinguishable at 91.8 °C and 121.2 °C; for the other flours, the second peak overlaps the first (93–103 °C), forming a shoulder at a higher temperature (122–134 °C) compared to the main peak [29,30]. 

The DSC analysis confirms that the start and end of the gelatinization/melting process depends on the amount and type of plasticizer. The presence of water anticipates and facilitates gelatinization by amorphizing portions of crystalline amylose, while the presence of glycerol, during the gelatinization/melting process, shifts the end of the gelatinization curve upwards in temperature, as already demonstrated [29,31]. The temperature of 150 °C and a residence time in the extruder not exceeding the DSC test time (15 min from 0 to 150 °C at 10 °C/min) are useful reference parameters for defining the plasticization process by melt compounding.

**Figure 4:**
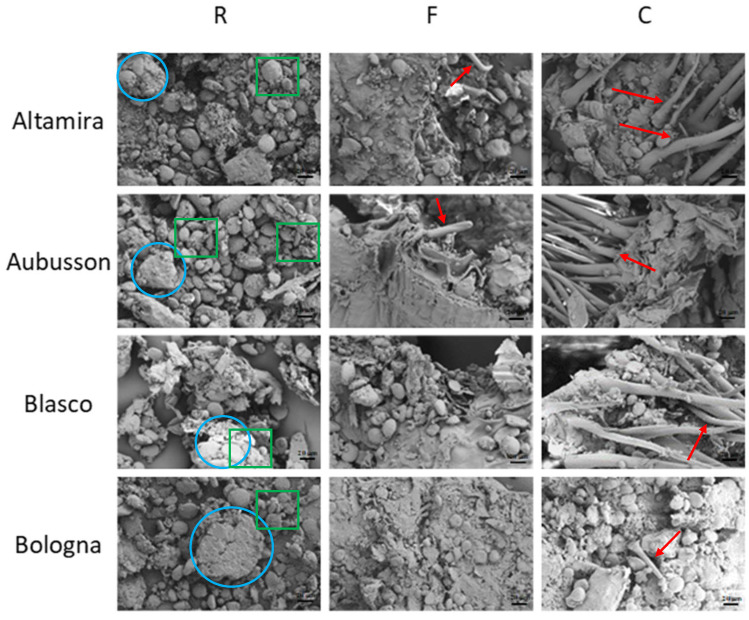
FESEM micrographs at 1.0 K magnification of one refined flour (R) and two wholegrain flours with fine (F) or coarse (C) grinding for each of the four bread wheat cultivars used for plasticization. (Starch granules = green squares; gluten = blue circles; bran fragments = red arrows).

Morphology: In Figure 4, SEM micrographs at 1.0 K show the refined (R) and wholegrain flours with fine (F) and coarse (C) grinding of the four cultivars. In the images of R flours, unfractionated starch conglomerates of granules appear to have a classic bimodal pattern, with large granules surrounded by small granules (green squares) [32]. These FESEM images highlight how the gluten, with a pasty appearance, surrounds the starch granules (blue circles) [4]. The protein matrix appears more compact and adherent to the starch in Blasco and Bologna [33]. It is noted that, in R flours, the bran content is very low and has smaller dimensions compared to wholegrain F flours. The difference appears even more evident when comparing R flour to wholegrain C flours, which show fragments of bran and brush hairs of the wheat grain measuring tens of micrometers (red arrows). The SEM images point out the morphological differences between the different milling levels and, in some aspects, between the flours obtained from different wheat varieties.

**Figure 5:**
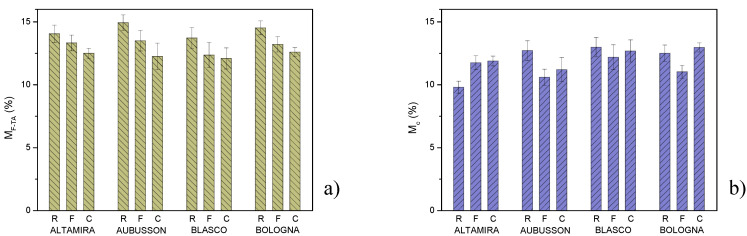
Moisture content, in controlled environmental conditions of 20 °C and 53% RH, for the samples (**a**) of flours and (**b**) of plasticized bulk samples obtained from the flours of four bread wheat varieties. The flours were refined (R) or wholegrain.

Moisture content: The moisture content of the flours was estimated from the mass loss at 170 °C in the TGA tests, assuming negligible other thermodegradative phenomena. The water absorbed in controlled environmental conditions (20 °C—53% RH) for R was between 13.7 ± 0.8 and 14.9 ± 0.6% wt., decreased to values between 12.4 ± 1.0 and 13.5 ± 0.8% wt. for wholegrain F flours and it was further reduced in the range 12.1 ± 0.8–12.6 ± 0.3% wt. for wholegrain C flours (Figure 5a). Therefore, the presence of bran appears to reduce the water absorption capacity, and the larger size of the bran fiber shows less propensity to imbibition compared to the fine-sized one [34,35]. 

Measurements of the moisture content of the plastic bulk samples report values between 9.8 ± 0.5 and 13.0 ± 0.8%wt. of water (Figure 5b). Altamira flour-based composites showed an increase in moisture content passing from the use of R flour to wholegrain flours. The moisture content for the plastic bulk samples from the R flours of the other three wheat varieties was higher than in case of Altamira, higher than for bulk samples from wholegrain F flours and comparable to that of bulk samples from wholegrain C flours. Differences in the moisture content of plastic samples between Altamira and the other three varieties are likely due to differences in protein and starch characteristics which affect the grinding outcome and the morphology of the interface between the plasticized matrix and the bran fiber [36].

**Figure 6:**
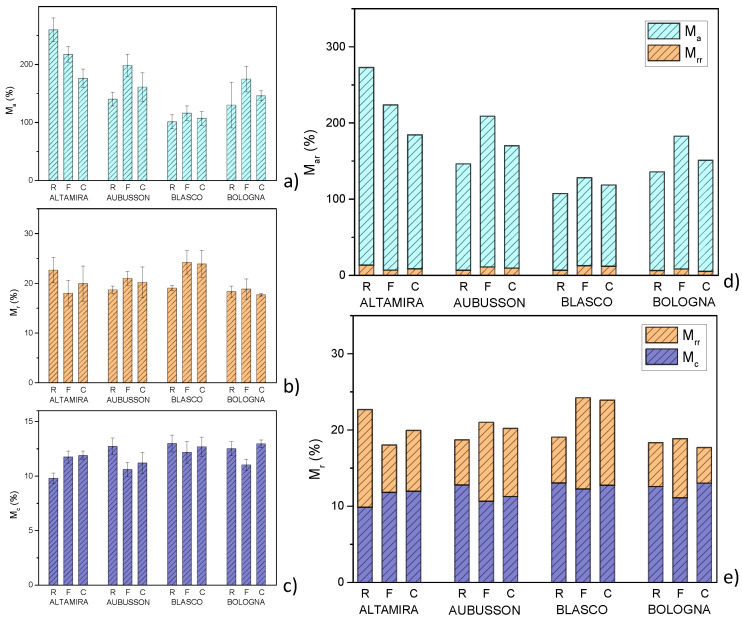
Measured values of: (**a**) water absorption, (**b**) mass loss and (**c**) moisture content of thermoplastics obtained from flours of four bread wheat cultivars. The flours were refined (R) or wholegrain with fine (F) or coarse (C) grinding. Values of: (**d**) real water absorption, (**e**) real mass loss calculated on the same samples.

Water absorption and mass loss of plasticized materials: In Figure 6, the behavior of the bulk samples placed in direct contact with water was evaluated in terms of water soaking and mass loss due to dissolution, release and detachment of material during immersion. The values (%wt.) of water absorption M_a_ and mass loss M_r_ after immersion for 24 h were measured. In Figure 6a, water absorption M_a_ is between 100% wt. of Blasco R and 260% wt. by Altamira R. Except for Altamira, the lowest absorption was obtained with the use of refined flours, increased with wholegrain F flours and decreased to intermediate values with wholegrain C flours. It was assumed that M_a_ depends on two antagonistic characteristics: the compactness of the plasticized matrix which hinders water absorption and the presence of discontinuities at the interface between the matrix and bran particles, which create preferential paths for the diffusion of humidity. Materials made from wholegrain F flours have a greater number of smaller particles, and therefore a more effective diffusion network, compared to those made from C flours. Differences in water absorption between plastics derived from Altamira and those derived from the other three varieties are probably related to differences in protein content and composition of flours. Although proteins might have a disruption effect on the inter-chain hydrogen bonds, Wang et al. [37] demonstrated that the increase in gluten content within certain limits leads to the establishment of self-similar structures with a higher density. The fractal dimension D obtained from the SAXS patterns carried out on plasticized wheat flours with different protein contents showed that the compactness increases with the gluten content. Greater compactness hinders the diffusion of water within the plasticized flours. 

In Figure 6b, the mass loss is between 17.6% wt. of Bologna C and 24.2% wt. of Blasco F. For the measurement method used, part of this mass loss consisted of the previously measured moisture content M_c_. In Figure 6d, the real water absorption M_ar_ was greater than the measured absorption M_a_ (it is necessary to take into account the share of real mass loss M_rr_, according to the equation M_ar_ = M_a_ + M_rr_). Real water absorption ranges from 107% to 273% with an average value of 169%. In Figure 6e, the mass loss consisted of the real mass loss to which the moisture content is added, i.e., M_r_ = M_rr_ + M_c_. The real mass loss was moderate, ranging between 4.7 and 12.8% by weight, with an average value around 8.3%. The water absorption and mass loss measurements emphasized the marked influence of environmental humidity on these materials, highlighting a weakness of materials based on plasticized flours. It should also be considered that the condition of immersion in water is extreme because such high humidity levels are rarely reached in the environment. However, the problem of the hydrophilicity of plasticized flour or starch-based materials, causing swelling and erosion, is limiting for various applications. Various solutions to the problem were proposed to make plasticized flours less hydrophilic [12,38,39,40].

**Figure 7:**
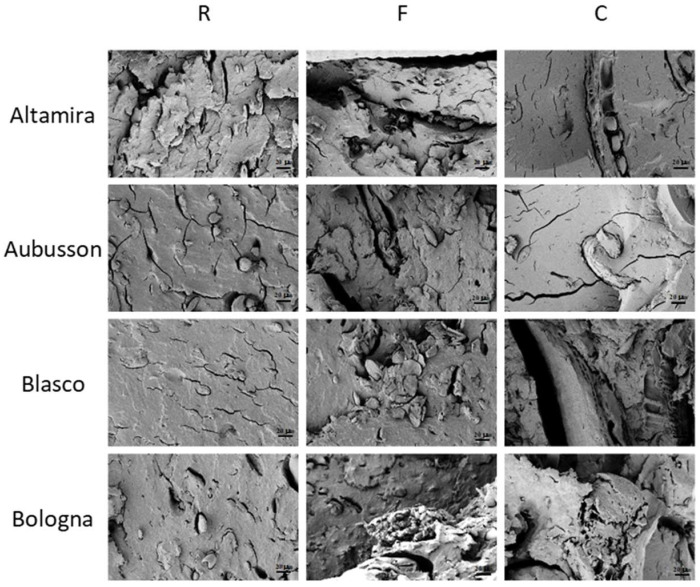
FESEM micrographs at 1.0 K magnification of the fractured surfaces of the thermoplastic bulk samples obtained from flours of four bread wheat cultivars. The flours were refined (R) or wholegrain with fine (F) or coarse (C) grinding.

Morphology of the plasticized materials: The morphologies of the fractured surfaces of the plasticized samples at 1.0 K of magnification were observed by FESEM (Figure 7) and differences were found for the flours from the four wheat varieties at a different milling level. In general, the plasticized samples from refined flours (Figure 7, column left) appeared well plasticized with a uniform surface and no evident bran particles can be noted. In detail, Altamira R shows a plasticized surface that is not very compact, with some cracks and no visible starch granules; the Aubusson R fracture surface showed well-plasticized results, with few cracks but with some separate starch granules. The Blasco R micrograph shows a compact surface, with some grooves and steps and only a few small cracks but no separated starch granules. The morphological aspect of the Bologna R fracture indicates a compact and well plasticized material, free of cracks and with some starch granules well interfaced with the matrix which, on a microscopic scale, resemble gravel aggregates.

In general, in the fracture surfaces of the samples obtained from plasticization of wholegrain flours F (Figure 7, center column), the bran fibers with a particle/lamellar appearance were uniformly distributed and well bonded to the plasticized starch, suggesting the realization of a composite materials with good characteristics [6,12,41]. The notations on the distribution of cracks already reported for the corresponding refined flours are also confirmed for these composites.

The morphological analysis of the composites obtained from wholegrain flours C (Figure 7, right column) highlights the presence of large particles and conglomerates of bran fibers, up to hundreds of microns, immersed in the plasticized matrix. The bond at the interface is generally good, although there are areas of partial detachment probably due to the great amount and size of the bran. This morphology suggests carrying out an evaluation between the effectiveness of the reinforcing function of the fibers and their weakening effect caused by the defects produced at the interface. In particular, Blasco C shows large areas of debonding between the reinforcement and the bioplastic matrix.

Mechanical characterization: The tensile tests carried out on the plasticized bulk specimens allowed the calculation of some mechanical parameters characteristic of the materials, such as Young’s modulus, tensile strength and elongation at break (Table 2).

**Figure 8:**
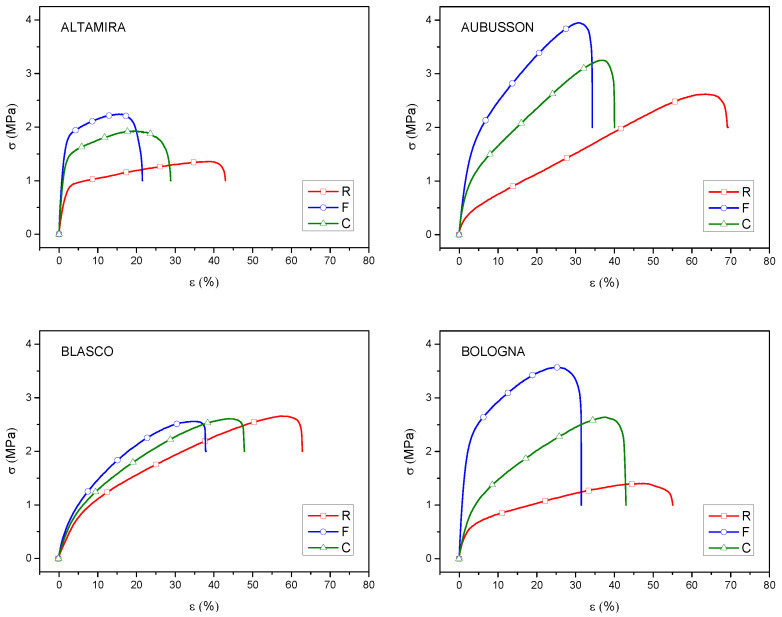
Stress–strain curves of the thermoplastic bulk samples obtained from flours of four bread wheat cultivars. The flours were refined (R) or wholegrain with fine (F) or coarse (C) grinding.

Some representative curves of the mechanical behavior of the specimens are also reported in Figure 8. In general, all thermoplastics bulk samples from refined flours were less resistant and more deformable than the corresponding samples from wholegrain flours. Furthermore, in samples from wholegrain flours of all varieties, the coarse bran particles increased the strength and decreased the deformability as compared to the fine bran. This result, if correlated to the morphological analysis carried out on the samples of wholegrain flour C, suggests that coarse bran particles cause, together with an effective fibrous reinforcement effect, also defects which constitute points of poor transmission of the matrix/reinforcement forces, delamination and therefore triggering of fractures, which compromise their real effectiveness. In general, the negative effect of defects due to the presence of bran fiber is much less evident for composites based on wholegrain F flours; this may be motivated by the smaller size of the bran particles, which are better dispersed and connected to the biopolymer matrix which covers them uniformly [6,12].

In general, the mechanical properties of these thermoplastic materials are satisfactory when compared to other biocomposites based on plasticized starch, in consideration of the economic and eco-sustainability advantages of wholegrain flours compared to refined starch [42]. Although the obtained values of s and e are in line with the expectations (as reported in our previous studies on mechanical performance of refined flours’ and bran’s role in improving tensile strength), comparison with more expensive and performing biocomposites based on neat synthetic polymers, as in the case of PLA, cannot be directly carried out [43]. Despite this general trend, some difference can be evidenced between thermoplastic bulk samples from different wheat varieties. In detail, the bulk samples obtained from Altamira showed low values of both tensile strength and elongation and break. On the contrary, both the strength and the elongation at break were higher in Aubusson, notwithstanding the soft-like baking properties of this variety. This behavior is confirmed by the morphological analysis, where good dispersion and effectiveness of the bond at the interface between matrix and fiber was noted. Bulk samples derived from Blasco R flour had good tensile strength and elongation at break but, differently from what observed for the other wheat varieties, the presence of bran as both fine and coarse particles did not improve the strength and only reduced deformability. Finally, the bulk samples from Bologna R flour showed low strength and high deformability, which was not expected, given the high value of W, while the presence of bran caused a relevant increase in strength, as for Altamira and Aubusson. The reasons for this different behavior of the plastics from these four varieties are not easy to explain but are likely related to differences in grain hardness and alveographic parameters [41,44], which give an overall information of different gluten composition (i.e., absolute and relative abundances of high and low molecular weight glutenin subunits and of gliadins) as hypothesized by Benincasa et al. [3,4]. Moreover, further information would be necessary on the starch abundance and starch granules size of different cultivars, and on the effect of different grain milling for R, and F and C flours of each variety on starch granules integrity. All these aspects will need to be further investigated in future studies on this subject.

**Figure 9:**
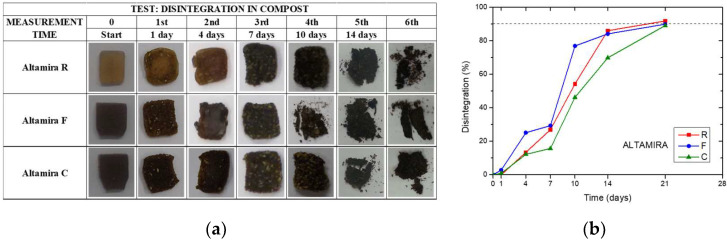
Visual images (**a**) and evolution of disintegration degree (**b**) at different compositing times for the thermoplastic bulk samples obtained from bread wheat flour of the variety Altamira refined (R) or wholegrain with fine (F) or coarse (C) grinding. Dashed line represents the disintegration threshold.

Degradation in simulated compost: Figure 9a shows the visual images of the thermoplastic samples for the three tested formulations during the progress of the disintegration process under composting conditions, while Figure 9b shows the trend of the mass disintegration rate in compost. All materials reached 90% disintegration after 21 days under composting conditions. Altamira C sample showed different disintegration kinetics, presenting lower disintegration values, in comparison with Altamira R and Altamira F. This behavior can be justified considering that greater bran particles slowed down the overall decomposition process of the plasticized fraction, due to the increased lignocellulosic content of such materials, that confers additional stability to the material [45]. On the other hand, fine grinding (F) accelerated the process, due to the observed morphology of the sample, creating the cracks responsible of water penetration during composting. Samples completely disintegrated within 21 days, confirming the compostability, at lab conditions, of these materials.

Phytotoxicity of compost: Results at 40 days after the beginning of composting (DABC) were affected by both the type of plastic and the concentration of compost extract (Table 3). All composts depressed the germination performance in terms of both the number of germinated seeds (GN) and the radicle length (L) of seedlings, and, in fact, the germination index (GI) was always below 70%. For all composts, the higher the extract concentration, the lower the GI value. The compost derived from refined flours gave the greatest phytotoxic effect, causing the lowest GI value. The presence of bran mitigated the phytotoxic effect, regardless of the bran grinding level; however, GI was still below 70%. 

On the contrary, at 60 DABC, all composts determined acceptable GI values (i.e., higher than 70%). In particular, GN approximated 100% and L was always similar to the control, except for the compost with plastics from R flours, where the radicle length was slightly reduced. The GI of 108% obtained in the case of the compost from whole grain flour F at the highest dilution of the extract might be the result of an hormetic effect. In fact, it is known in the literature that several synthetic or natural substances (e.g., NaCl and other salts, herbicides, allelopathic substances, etc.), which generally depress plant growth, may instead have a stimulatory effect when used at very low concentrations [46,47]. Based on these results, it can be concluded that a complete maturation of the compost, able to guarantee no phytotoxic effects, requires a time interval of around 2 months. 

## 4. Conclusions

Results indicate that thermoplastic bulk samples obtained from wholegrain bread wheat flours had greater tensile strength and lower elongation at break than the bulk samples obtained from refined flours. The effect was clearly due to the presence of bran and was greater with fine than with coarse grinding. This result can be generalized, since it stands for flours from each of the four wheat varieties used in this experiment, which were very different in grain hardness and baking properties (as indicated by the values of Chopin’s alveograph parameters), revealing a different composition in gluten fractions. The reasons for the effect of bran and grinding levels have been discussed based also on the SEM microscopy of samples, which reveals that the bran works as a reinforcement and affects starch granules exposure to plasticizer. In the case of water absorption, it has been found that the lowest value was obtained with the use of refined flours, and increased with wholegrain F flours and decreased to intermediate values with wholegrain C flours, emphasizing again the role of the bran grinding: compactness of the plasticized matrix hinders water absorption, and, on the other hand, the presence of discontinuities at the interface between the matrix and bran particles created preferential paths for the diffusion of humidity. The bran also affected the plastic decomposition in compost, in particular the fine grinding accelerated the decomposition, while the coarse grinding slowed it as compared to the decomposition observed in plastics obtained from refined flours. The use of wholegrain wheat flours and the grinding level represent novel choice factors to consider for tailoring thermoplastic manufactures according to requirements and uses. Furthermore, this research is indeed needed to understand the role of protein and starch characteristics, as affected by the wheat variety and cultivation practices, on the mechanical properties of derived thermoplastic materials. The results of this investigation will be useful to properly design new plastic formulations requiring different mechanical performance, by addressing the choice on wheat flours that can differ for grain hardness, alveographic parameters, bran presence and grinding level.

## Figures and Tables

**Table 1:** Grain hardness and protein content, and Chopin’s alveograph parameters of refined flours from the four bread wheat cultivars used for plasticization.

Wheat Flour	Grain	Protein	W	P	L	P/L
Name	Hardness	% wt.	(10^−4^ J)	(mm_H2O_)	mm	a.u.
Altamira	Soft	12.0	144	43	121	0.36
Aubusson	Hard	13.3	160	41	196	0.21
Blasco	Hard	13.6	355	94	113	0.83
Bologna	Hard	13.7	360	71	150	0.47

**Table 2:** Young’s modulus (E), tensile strength (σ) and strain at break (ε) of thermoplastic bulk samples obtained from flours of four bread wheat cultivars. The flours were refined (R) or wholegrain with fine (F) or coarse (C) grinding.

Flour/Milling Level		Young’s Modulus	Tensile Strength	Elongation at Break
		MPa	MPa	%
	R	115± 11	1.3 ± 0.1	43.6 ± 1.9
Altamira	F	139 ± 5	1.9 ± 0.1	23.5 ± 3.7
	C	146 ± 6	1.8 ± 0.1	27.9 ± 1.3
	R	30 ± 4	2.4 ± 0.2	66.8 ± 2.7
Aubusson	F	94 ± 13	3.8 ± 0.2	33.8 ± 1.4
	C	59 ± 16	3.1 ± 0.1	38.9 ± 1.0
	R	41 ± 4	3.5 ± 0.2	66.3 ± 11.4
Blasco	F	33 ± 3	2.5 ± 0.3	43.5 ± 3.1
	C	26 ± 9	2.6 ± 0.4	47.5 ± 9.8
	R	36 ± 1	2.2 ± 0.4	54.2 ± 17.3
Bologna	F	156 ± 5	3.8 ± 0.2	33.3 ± 3.6
	C	59 ± 16	2.6 ± 0.1	43.5 ± 1.0

**Table 3:** Germination performance of cress seeds on compost extracts at 50% and 75% concentration in water (*w*:*w*), and at 40 and 60 days after the beginning of composting (DABC). Composts were obtained from thermoplastics derived from bread wheat flours refined (R) or wholegrain with fine (F) or coarse (C) grinding. GN: average number of germinated seeds; L: seedling length; GI: germination index.

Flour Type	Dilution	40 DABC		60 DABC	
		GNxL	GI%	GNxL	GI%
Control	-	37.0		233	
R	50%	9.5	26	183	79
F	50%	21.0	57	251	108
C	50%	20.0	54	229	98
R	75%	1.9	5	171	73
F	75%	12.5	34	213	91
C	75%	13.3	36	197	85

## Data Availability

The data presented in this study are available on request from the corresponding author.
